# How do world class top 5 Giro d'Italia finishers train? A qualitative multiple case study

**DOI:** 10.1111/sms.14201

**Published:** 2022-06-18

**Authors:** Gabriele Gallo, Manuel Mateo‐March, Daniel Gotti, Emanuela Faelli, Piero Ruggeri, Roberto Codella, Luca Filipas

**Affiliations:** ^1^ Department of Neuroscience, Rehabilitation, Ophthalmology, Genetics, Maternal and Child Health University of Genoa Genoa Italy; ^2^ Centro Polifunzionale di Scienze Motorie University of Genoa Genoa Italy; ^3^ Sport Science Department Miguel Hernández University of Elche Madrid Spain; ^4^ Faculty of Sport Sciences European University of Madrid Madrid Spain; ^5^ Department of Biomedical Sciences for Health Università degli Studi di Milano Milan Italy; ^6^ Department of Experimental Medicine University of Genoa Genoa Italy; ^7^ Department of Endocrinology, Nutrition and Metabolic Diseases, IRCCS MultiMedica Milan Italy

**Keywords:** case study, elite cyclists, general classification, performance, stage race

## Abstract

The aim of this study was to describe individual training strategies in preparation to Giro d'Italia of three world class road cyclists who achieved a top 5 in the general classification. Day‐to‐day power meter training and racing data of three road cyclists (age: 26, 27, 25 years; relative maximum oxygen consumption: 81, 82, 80 ml·min^−1^·kg^−1^; relative 20‐min record power output: 6.6, 6.6, 6.4 W kg^−1^) of the 22 weeks (December–May) leading up to the top 5 in Giro d'Italia general classification were retrospectively analyzed. Weekly volume and intensity distribution were considered. Cyclists completed 17, 22, 29 races, trained averagely for 19.7 (7.9), 16.2 (7.0), 14.7 (6.2) hours per week, with a training intensity distribution of 91.3–6.5‐2.2, 83.6–10.6‐5.8, 86.7–8.9‐4.4 in zone 1‐zone 2‐zone 3 before the Giro d'Italia. Two cyclists spent 55 and 39 days at altitude, one did not attend any altitude camp. Cyclists adopted an overall pyramidal intensity distribution with a relevant increase in high‐intensity volume and polarization index in races weeks. Tapering phases seem to be dictated by race schedule instead of literature prescription, with no strength training performed by the three cyclists throughout the entire periodization.

## 1 INTRODUCTION

Road cycling is one of the most extreme endurance sports having the highest exercise volume per year (~30 000–35 000 km, ~1000 h)^1^ and some of the most demanding competitions such as 3‐week Grand Tours (Giro d'Italia, Tour de France, Vuelta a España), in which athletes compete for 21 days (~100 h) with only two rest days in between. In the last years, there has been a rapid increase in the capacity to capture real‐time data through portable mechanical power meters. This has led to a deeper understanding of both race demands^2^, ^3^, ^4^ and parameters associated with competition outcomes in road cycling.^4^, ^5^ However, there is a paucity of study reporting training characteristics of professional road cyclists with only few studies reporting the professional cyclists training data as a mean of the sample considered.^6^, ^7^, ^8^ To the best of our knowledge, no study has reported the individual day‐to‐day training and racing data of world‐class road cyclists preceding an important competition, as it has already been done in recent studies in world class endurance athletes.^9^, ^10^ Reporting this data could be relevant for coaches and practitioners to compare world‐class athletes' preparation strategies with those outlined by scientific research in controlled studies. This could provide useful insights to: (i) highlight whether literature's “best practice” is adopted or not by world class road cyclists; (ii) acknowledge training strategies adopted by world class road cyclists to be tested in future scientific studies. Therefore, the aim of this study was to describe the preparation strategies leading to a top 5 position? in the general classification of Giro d'Italia of three world class road cyclists.

## 2 MATERIALS AND METHODS

### 2.1 Participants

Three professional road cyclists were included in this study. Their anthropometric and physiological characteristics are reported in Table 1. They can be classified as “world class” according to McKay's participant classification framework for research in sport science.^11^ The study design and procedures were approved by the research ethics committee of the Università degli Studi di Milano (approval number 52/20, attachment 4) and followed the ethical principles for medical research involving human participants set by the World Medical Association Declaration of Helsinki. Participants gave informed written consent.

**TABLE 1:** Anthropometric and physiological characteristics of the three cyclists

	Cyclist A	Cyclist B	Cyclist C
Age (years)	26	27	25
Weight (kg)	64	57	61
Height (cm)	173	166	170
Relative maximum oxygen consumption (ml·min^−1^·kg^−1^)	81	82	80
20‐min record power output (W)	420	378	389
Relative 20‐min record power output (W·kg^−1^)	6.6	6.6	6.4
20‐min record power output after 45 kJ·kg^−1^ (W)	420	367	379
Relative 20‐min record power output after 45 kJ·kg^−1^ (W·kg^−1^)	6.6	6.4	6.2

### 2.2 Experimental design

For each of the three cyclists (cyclist A, cyclist B, cyclist C), training and racing data of the 22 weeks preceding the beginning of the Giro d'Italia in which they achieved the top 5 positions in the general classification were considered. The seasons corresponding to this achievement were between 2015 and 2018. As the Giro d'Italia starts in the first week of May, the time frame analyzed started approximately from December of the year before (i.e., beginning of the preparation phase). Weekly training and races power data were considered starting from Monday of the first week of the preparation period, except for tapering analysis. Since the Giro starts on Friday/Saturday, the last week included the first days of the Grand Tour. For tapering analysis, the weeks were counted starting from the day before the official start of Giro d'Italia, considering only the final 6 weeks preceding the Grand Tour. Race schedule of each athlete was reported. Races were classified based on relative importance for that rider (i.e., A, main goal; B, secondary goal; C, preparation race), days of race and level of the race according to the Union Cycliste Internationale classification. The altitude training camp or period was also reported.

### 2.3 Data processing

Power output from training and races was daily collected using portable power meters (cyclist A: Power2max, Saxonar GmbH, Waldhufen; cyclists B and C: SRM GmbH, Jülich, Germany) that were zeroed before every ride. The accuracy of these power meters was reported in a previous study.^12^ Data were saved and organized using a cycling performance software analyzer (WKO5; TrainingPeaks LLC. All data were visually checked for erroneous data and incomplete data files, due to technological issues (e.g., flat battery of a power meter), were removed when necessary. The inclusion criteria for power data were that the weekly cycling volume was at least 80% of the weekly total volume. This cutoff was chosen in order to have a plausible weekly intensity distribution. Only two weeks of cyclists B (Week 9 and Week 12) did not meet the inclusion criteria and so were excluded from the analysis. When the weekly cycling volume was >80% but did not reach 100% of the weekly total volume, power parameters were increased proportionally. For cyclist B, volume but not power data were reported for the first 4 weeks of preparation (Weeks 49, 50, 51, and 52) as he trained using a mountain bike without power meter during that period.

### 2.4 Volume and intensity distribution

Volume was considered as duration of the training/racing session. Intensity distribution was calculated using a three‐zone power‐based model: functional threshold power^13^ was used to separate zone 2 and zone 3 as it is a valid surrogate of lactate threshold.^14^ Functional threshold power was defined as the highest power output a cyclist can maintain in a quasi‐steady state for approximately 60 min^15^; it was estimated by subtracting the 5% to the highest mean of 20‐min power output recorded in race or training^13^ and was updated on an annual basis. The 85% of the functional threshold power was used to separate zone 1 and zone 2 because it represents an exercise intensity close to the first lactate/ventilatory threshold in professional cyclists.^2^, ^16^ Time spent in zone 1 was considered as low intensity training (LIT), time in zone 2 as medium intensity training (MIT), and time in zone 3 as high intensity training (HIT). The polarization index^17^ was also used to describe the training intensity distribution adopted by the three cyclists. Polarization index has the advantage of summarizing in a single variable the nature of the intensity distribution and has been recently used for this purpose.^18^ If polarization index was >2.00, the training intensity distribution was defined as “polarized,” while with polarization index ≤2.00, the training intensity distribution was defined as non‐polarized.

### 2.5 Statistical analysis

A descriptive statistic for each athlete was used. For each variable individual mean, standard deviation, maximum and minimum, and weekly values were reported. In the tapering analysis, the differences in volume and intensity distribution between Week −2 and Week −1 compared with Weeks −3/−6 were calculated. Intensity distribution training and racing data were analyzed both aggregated and separated.

## 3 RESULTS

### 3.1 Volume and intensity distribution

**FIGURE 1:**
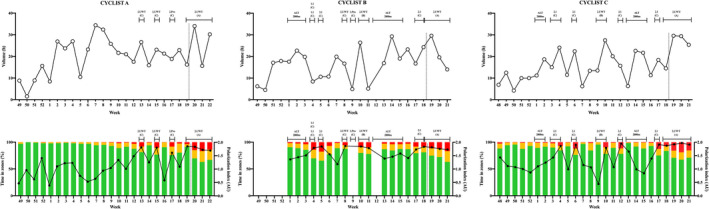
Weekly volume, intensity distribution and polarization index of the three cyclists in preparation to Giro d'Italia. Dot line marks the start of the Giro d'Italia. Altitude camps (ALT) and race type and priority are reported above each graph

**FIGURE 2:**
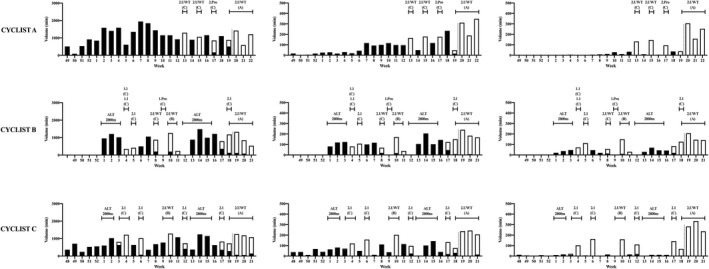
Training (black box) and races (white box) volumes at low intensity (left graphs), medium intensity (center graphs), and high intensity (right graphs) for the three cyclists in preparation to Giro d'Italia. Dot line marks the start of the Giro d'Italia. Altitude camps (ALT) and race type and priority are reported above each graph

Training and races characteristics of the three cyclists are reported in Table 2. Weekly volume, training and races intensity distribution, and polarization index of the three cyclists are shown in Figures 1 and 2.

**TABLE 2:** Training and races characteristics of the three cyclists in preparation to the Grand Tour

	Cyclist A	Cyclist B	Cyclist C
Race days			
Total days (*n*)	17	22	29
Days in stage races (*n*)	17	19	29
Days in one day races (*n*)	0	3	0
Altitude camps			
Total days (*n*)	0	55	39
Camps (*n*)	0	2	2
Weekly volume			
Mean hours (SD)	19.7 (7.9)	16.2 (7.0)	14.7 (6.2)
Maximum volume (h)	34.4	29.3	27.7
Minimum volume (h)	1.5	4.5	4.2
Training‐race proportion (%)	78.3–21.7	69.9–30.1	66.9–33.1
Weekly low‐intensity training			
Mean hours (SD)	1077 (445)	908 (349)	764 (297)
Maximum volume (min)	1942	1484	1288
Minimum volume (min)	90	238	240
Training‐race proportion (%)	87.6–12.4	70.9–29.1	72.9–27.1
Weekly medium‐intensity training			
Mean hours (SD)	77 (67)	115 (39)	78 (50)
Maximum volume (min)	233	205	203
Minimum volume (min)	0	38	5
Training‐race proportion (%)	69.7–30.3	64.7–35.3	60.6–39.4
Weekly high‐intensity training			
Mean hours (SD)	25 (41)	62 (37)	39 (54)
Maximum volume (min)	146	149	163
Minimum volume (min)	0	20	1
Training‐race proportion (%)	29.6–70.4	40.8–59.2	18.3–81.7
Total volume			
Low‐intensity training (%)	91.3	83.6	86.7
Medium‐intensity training (%)	6.5	10.6	8.9
High‐intensity training (%)	2.2	5.8	4.4
Polarization index (AU)	1.47	1.65	1.63
Training volume			
Low‐intensity training (%)	93.9	86.6	91.0
Medium‐intensity training (%)	5.3	10.0	7.8
High‐intensity training (%)	0.8	3.4	1.2
Polarization index (AU)	1.15	1.46	1.14
Race volume			
Low‐intensity training (%)	78.0	77.3	76.7
Medium‐intensity training (%)	12.6	11.9	11.5
High‐intensity training (%)	9.4	10.8	11.8
Polarization index (AU)	1.76	1.84	1.89

### 3.2 Tapering

**FIGURE 3:**
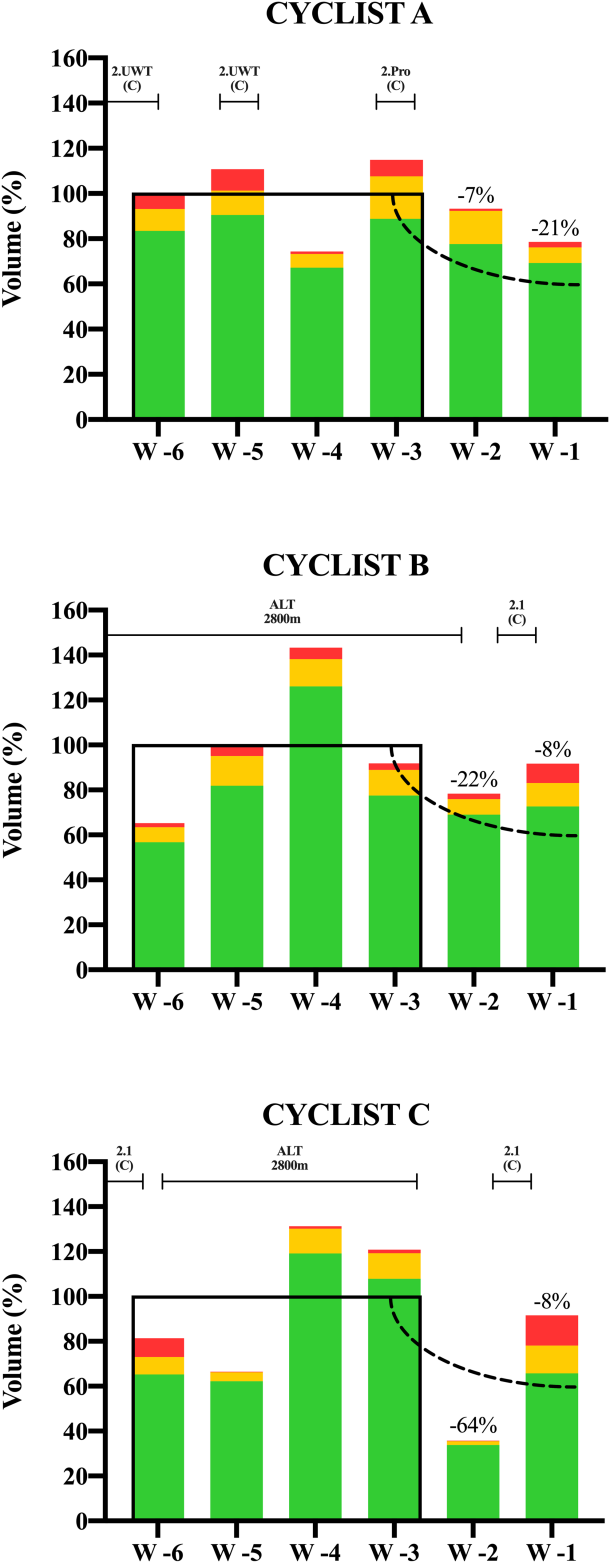
Volume and intensity distribution during the 6 weeks preceding the Giro d'Italia. Black lines represent the average volume for Weeks −3/−6; dot lines represent the suggested taper based on the literature recommendations. Altitude camps (ALT) and race type and priority are reported above each graph

Volume and intensity distribution in Week −2 and Week −1 compared with Weeks −3/−6 are reported in Figure 3.

## 4 DISCUSSION

### 4.1 Volume

The world class cyclists' average weekly volume in preparation to the top 5 achievement in the general classification of Giro d'Italia was not higher compared with the average weekly training volume over an entire season reported in a cohort of 20 World Tour professional road cyclists.^8^ Therefore, the three cyclists did not adopt an exceptional average weekly volume in preparation to their top 5 achievement. This is in line with the fact that even if training volume is considered a key factor of endurance training,^19^ increasing already high volume for long periods did not seem to further increase performance.^20^, ^21^

### 4.2 Intensity distribution

The overall intensity distribution of the entire preparation period was pyramidal in all the three cyclists with a large proportion of high‐volume low intensity training. This means that most of the time was spent at low intensity, and a decreasing proportion of time at medium and high intensity. This is in line with previous studies reporting intensity distribution over long periods (i.e., >8 weeks) in elite endurance athletes.^6^, ^10^ However, randomized controlled trial studies conducted on non‐elite subjects lasting 6–10 weeks reported superior responses to polarized especially when compared with pyramidal or threshold intensity distribution.^22^, ^23^, ^24^ Furthermore, it has been shown that polarized intensity distribution is a strategy adopted by successful elite athletes during the competition phase.^24^ Regarding this aspect, when looking at Figure 1, it is easy to understand that during the racing weeks the intensity distribution tends to be more polarized compared with training weeks in all the three cyclists. This is confirmed by the higher polarization index in races compared to training (Table 2). The higher polarization index in race weeks derived from a huge increase of high‐intensity volume (Figure 1 and Figure 2) which could be due to the competitive nature of races. While it is well‐known that high‐intensity training leads to rapid adaptions of various tissues and to an increase of aerobic and anaerobic performance indexes in less time compared with other exercise intensities,^25^, ^26^ a huge amount of high‐intensity volume over several weeks leads to no further adaptations^23^, ^27^ and might even lead to distress symptoms.^19^, ^24^ This could be one of the reasons contributing to the high‐volume pyramidal distribution observed in the training of all the three cyclists. Furthermore, no previous randomized controlled trials have investigated the effect of intensity distribution on fatigue resistance, a key determinant of competition outcomes in under 23 and professional road cyclists,^4^ which could be potentially responsive to volume at low intensity.^28^ Regarding this aspect, it is noteworthy to highlight that the decay in 20‐min record power output after 45 kJ kg^−1^ of the athlete considered in this study are in line with the top 10–25 percentile of the normative data of World Tour cyclists presented in a recent study.^29^

### 4.3 Periodization

The three cyclists seem to have large variations both in volume and training intensity distribution over time, which is in line with previous studies suggesting that when the exercise load is high (as in world class athletes), training variations instead of training monotony is a superior strategy to gain fitness while avoiding distress symptoms.^30^ The only visible common pattern between the three cyclists is a clearly remarkable increase in volume at high‐intensity and polarization index in races weeks when compared with training weeks. Since these variations depend on when races are allocated, we could identify a sort of “high‐intensity races‐based block periodization.” The rationale of block periodization is that variations in training stimuli hypothetically avoid stagnation and lack of responsiveness in the molecular adaptive response. A series of studies of Rønnestad and colleagues showed that high‐intensity block periodization leads to superior improvement in well‐trained cyclist when compared with high‐intensity evenly distributed across 4–12 weeks.^31^, ^32^ However, a recent study showed similar improvements between block and traditional periodization over 12 week in trained cyclists.^33^ This is line with the case study of Solli and colleagues,^34^ reporting that the world's best cross‐country skier succeeded using both traditional and block periodization during her career. In the present study, the three cyclists seem to adopt block periodization caused by the choice of secondary goals and preparation races before the Giro d'Italia. This race scheduling has been chosen as they reported high intensity to be less psychologically demanding when performed during races compared with training sessions, as the presence of competitors enhances motivation and reduces perception of effort. The fact that stage races were preferred to one day races (Table 2) is linked to the nature of Giro d'Italia that involves a 3‐week stage race, where the key factor of race result is recovery. Future controlled studies should investigate whether consecutive “hard days” are or not actually the best strategy to improve recovery. Interestingly, while cyclist B and C raced already in the first part of the preparation (January), cyclist A raced for the first time at Week 13 (April). As a consequence, while cyclists B and C used block periodization throughout all the preparation, cyclists A first performed a high‐volume pyramidal periodization phase in which he progressively increased volume (Weeks 49–7), than he reduced volume and increased medium and high‐intensity and polarization progressively (week 8–12) until the first competition phase (Figure 1 and Figure 2) Basically, he followed a linear periodization, switching gradually from a pyramidal to a more polarized intensity distribution. Recently, Filipas et al.^18^ reported that a pyramidal to polarized periodization could be superior than a pyramidal or polarized one in well‐trained runners over a 16 week period. However, to the best of our knowledge, no studies have investigated the effect of combining sequentially linear and block periodization compared to only block or traditional one. Therefore, we could not give definitive recommendations whether insert or not races far from the main competition goal is or not the best preparation strategy. We could only acknowledge that cyclist A obtained his best general classification result in a 3‐week Grand Tour in the season analyzed, while this was not the case for cyclist B and cyclist C. This transition from linear to block periodization should be tested in future control‐studies to check whether it can provide higher adaptations. In general, periodization is probably linked to the training status of the athlete, with daily/weekly changes in volumes and intensities based on the training and wellness monitoring.

### 4.4 Tapering

The current literature's recommendations on tapering suggest a volume reduction of 40%–60% in the last 7–14 days before the main competition goal to maximize performance gains.^35^ Despite this scientific evidence, the three world class cyclists adopted a volume reduction of 7%, 22%, 64%, and 21%, 8%, 8% (Figure 3) during the last 2 weeks before Giro d'Italia, substantially less than the suggested reduction. However, this approach is in line with Tønnessen and colleagues,^36^ which found only a 5% and 15% decrease in volume during the last 2 weeks before a gold medal in elite cross‐country skiers and biathletes, respectively. Cyclist B and C performed a stage race in the final 7 days prior to Giro d'Italia: they followed a sort of inverse tapering of volume (i.e., volume Week −1 > volume Week −2) with a massive increase in high‐intensity volume in Week −1 compared with both Week −2 and the pre‐taper period. There is strong evidence that high‐intensity volume is a key factor to maintain and enhance physiological and performance adaptations during the taper phase.^37^ However, while the general literature's recommendation is to maintain intensity during taper,^35^ cyclists B and C even increased high‐intensity volume during Week −1. Regarding this aspect, we must acknowledge that the maintenance of the intensity referred to studies in which intensity was usually already high in the weeks before taper. This is not the case of cyclists B and C as the pre‐taper period corresponded almost entirely to high‐volume low‐intensity altitude training. This particular taper strategy (i.e., a races week with a huge increase in high‐intensity volume during taper after ~4 weeks of altitude training in pre‐taper) could theoretically be very effective as it combines the high‐intensity fast adaptative stimulus with the altitude training hematological adaptations.

On the contrary, the cons of this taper strategy could be that performing a stage race with only 3–4 days of recovery before a 3‐week Grand Tour could lead to progressive fatigue in the early phases of the Giro d'Italia.

### 4.5 Altitude training

While cyclist A did not perform altitude training throughout all the preparation, cyclists B and C performed two altitude training periods before Giro d'italia, which is line with the suggestion to perform multiple altitude periods over one year in order to maximize positive benefits.^38^ The duration of each altitude period (i.e., 2–4 weeks) is in line with literature recommendations.^39^ The altitude above the sea level was ~2800 m, slightly above the 1800–2500 m range suggested.^40^ Even if higher altitude has been proposed and this might cause overtraining and distress symptoms, the fact that cyclists B and C are altitude natives could have ameliorated the negative effect of high‐altitude exposure. The two cyclists followed the classical altitude training, which consists of sleeping and training at the same altitude. During altitude training, the intensity distribution was pyramidal focusing on high‐volume low‐ and medium‐intensity: this is in line with the literature's suggestion to exploit the hypoxic stimulus fostering high‐volume low‐intensity training rather than accumulating high‐intensity training.^40^ Interestingly, cyclists B and C adopted the same type of altitude periodization (i.e., the first altitude training period in the first part of preparation and the second finishing only 2 weeks before the start of Giro d'Italia). Furthermore, one or two weeks after the altitude training camp, both cyclist B and C raced, and this corresponded to a huge increase in high‐intensity volume and a more polarized intensity distribution. As explained above for taper, this could be an effective strategy to combine the positive adaptations obtained through altitude training and high‐intensity block periodization.

### 4.6 Strength training

None of the three cyclists performed strength training during the period analyzed. This is in contrast with previous studies which showed superior physiological and performance improvements in well‐trained and elite road cyclists after 10–25 weeks of heavy strength training.^41^ The reason why strength training was not performed was that the three cyclists were unwilling to perform strength training despite the coaches' indications. Whether a better persuasion's method could have led to a better race result remains unknown given the descriptive nature of this study.

## 5 PERSPECTIVE

In professional road cyclists, race scheduling (especially stage races) during the preparation period seems to be the pivotal factor determining periodization, as races weeks correspond to a huge increase in high‐intensity volume and polarization index. Tapering should be carefully customized based on athletes' races schedule and willingness to train at altitude.

In conclusion, in the preparation period leading to a top 5 in the general classification of Giro d'Italia, three world class road cyclists did not adopt a particularly higher weekly training volume compared to traditional elite cyclists' season training volume. Despite some inter‐individual differences, they adopted an overall pyramidal intensity distribution and a “high‐intensity races‐based block periodization” (i.e., a greater increase in high‐intensity volume and polarization index in races weeks). While one cyclist did not perform altitude training, the other two cyclists adopted similar altitude training strategies that are generally in line with literature's evidence. Taper strategies were substantially different from literature's best practice with less volume reduction than recommended, with two out of three cyclists performing a stage race in the last week before Giro d'Italia after 4 weeks of altitude training. Despite literature's recommendations, cyclists did not perform strength training.

## CONFLICT OF INTEREST

The authors declare no conflict of interests.

## Data Availability

The data that support the findings of this study are available on request from the corresponding author. The data are not publicly available due to privacy or ethical restrictions.
